# Excitation–Emission Matrix Fluorescence Spectroscopy Coupled with PARAFAC Modeling for Viability Prediction of Cells

**DOI:** 10.1021/acsomega.2c05383

**Published:** 2023-04-27

**Authors:** Klaudia Głowacz, Sandra Skorupska, Ilona Grabowska-Jadach, Rasmus Bro, Patrycja Ciosek-Skibińska

**Affiliations:** † Chair of Medical Biotechnology, Faculty of Chemistry, Warsaw University of Technology, Noakowskiego 3, 00-664 Warsaw, Poland; ‡ Department of Food Science, University of Copenhagen, Rolighedsvej 30, DK-1958 Frederiksberg C, Denmark

## Abstract

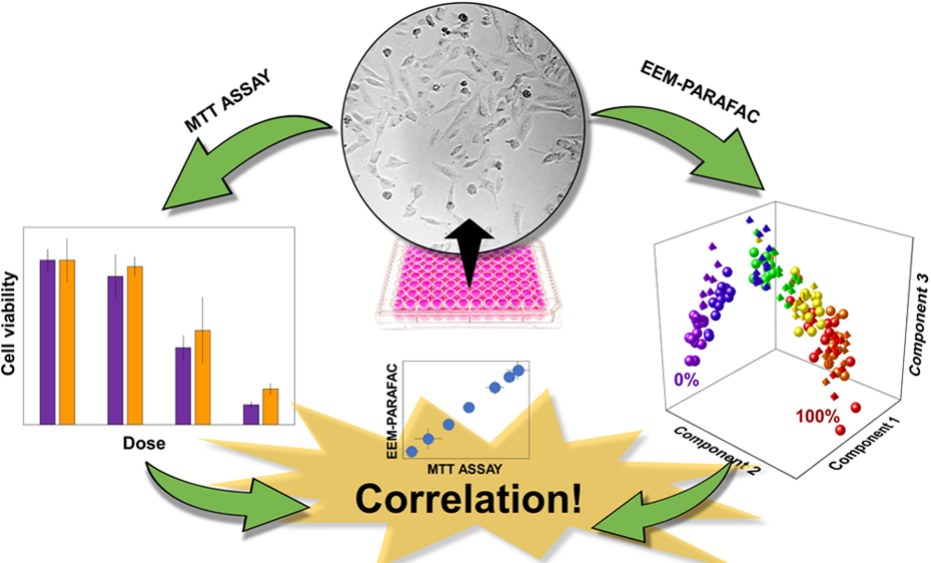

Cell-based sensors and assays have great potential in bioanalysis, drug discovery screening, and biochemical mechanisms research. The cell viability tests should be fast, safe, reliable, and time- and cost-effective. Although methods stated as “gold standards”, such as MTT, XTT, and LDH assays, usually fulfill these assumptions, they also show some limitations. They can be time-consuming, labor-intensive, and prone to errors and interference. Moreover, they do not enable the observation of the cell viability changes in real-time, continuously, and nondestructively. Therefore, we propose an alternative method of viability testing: native excitation–emission matrix fluorescence spectroscopy coupled with parallel factor analysis (PARAFAC), which is especially advantageous for cell monitoring due to its noninvasiveness and nondestructiveness and because there is no need for labeling and sample preparation. We demonstrate that our approach provides accurate results with even better sensitivity than the standard MTT test. With PARAFAC, it is possible to study the mechanism of the observed cell viability changes, which can be directly linked to increasing/decreasing fluorophores in the cell culture medium. The resulting parameters of the PARAFAC model are also helpful in establishing a reliable regression model for accurate and precise determination of the viability in A375 and HaCaT-adherent cell cultures treated with oxaliplatin.

## Introduction

Maintaining cells *in vitro* for biopharmaceutical production and conducting research using cell cultures requires their monitoring, usually performed by traditionally established, standard methods, such as differential staining, metabolic activity assays, or flow cytometry. Most of these methods provide exact but incomplete information on the cell state. They lack the ability to assess the information in real-time and noninvasively, thus tracing changes without terminating the experiment.^1^ Therefore, many alternative approaches have been proposed, including electrochemical techniques, such as impedance, conductometry, amperometry, and various optical sensors and biosensors.^2^ Electromagnetic spectroscopic techniques coupled with chemometric data analysis can also be applied, which is especially advantageous due to their noninvasiveness.^3^ On an industrial scale, for many years, various types of spectroscopies were used to determine multiple cell cultivation parameters^4^ such as concentrations of ammonium, glucose, glutamine, lactate, or total cell density (accessible by near-infrared (NIR) spectroscopy); acetic acid, antibodies, ethanol, fructose, glucose, lactate (accessible by mid-infrared (MIR) spectroscopy); ATP, ethanol, NAD(P)H, proteins, pyruvate, total cell density, vitamins (accessible by fluorescence spectroscopy); and acetate, glutamine, glutamate, total cell density (accessible by Raman spectroscopy^5^). The determination of the viability of cells is more demanding, and spectroscopic sensors are still in the research stage; however, recently, NIR and Raman spectroscopy to determine the viable cell count was proposed.^6^

While Raman spectroscopy^7−9^ and infrared (IR) spectroscopy^10,11^ used to be most popular in such applications, excitation–emission matrix fluorescence spectroscopy (EEM fluorescence spectroscopy, 2D fluorescence) seems to be a promising technique due to the possibility of very sensitive detection and quantification of various fluorophores present in *in vitro* cultures. EEM fluorescence spectroscopy is a modern, noninvasive analytical technique based on acquiring emission spectra at multiple excitation wavelengths.^12^ The result of such a measurement can be presented in the form of a 2D fluorescence landscape (excitation–emission matrix, EEM), regarded as a characteristic and unique signature of the tested sample (“fingerprint”), which encodes information on the fluorescent substances present in it, in terms of their type, concentration, and interactions between all the sample’s constituents. This complex information must be decoded using various numerical methods based on chemometric modeling and machine learning. Until now, EEM fluorescence spectroscopy has been used extensively, e.g., for bioprocess monitoring, quality control of foodstuff, and tracking of pollutants in water samples.^2,13−19^ It has also been shown that EEM fluorescence spectroscopy can detect the viable cell density; however, this observation was based on monitoring only one cell type (Chinese hamster ovary cell).^20,21^

In our previous research, we focused on developing many electrochemical multisensory systems (so-called “electronic tongues”), including an electrochemical imaging system used to monitor cell cultures exposed to various stress factors and enable the detection of the cytotoxic effect of 5-fluorouracil on A549 cells.^17^ Despite its noninvasiveness and quite simple measurement procedure, it presents some drawbacks due to the applied sensing strategy, i.e., the application of chemical sensors (lifetime, calibration frequency, deterioration of operational parameters). EEM fluorescence spectroscopy does not show any of these limitations; therefore, we attempted to use it for cell culture monitoring and compared it with the sequential injection analysis (SIA) electronic tongue.^22^ We showed that both approaches detected the cytotoxic effect after incubating A549 and MRC-5 cells with diclofenac sodium salt. Moreover, both systems determined incubation time and amounts of necrotic cells with high accuracy and precision. Following these promising results, we decided to study more in depth the possibility of applying EEM fluorescence spectroscopy for viability determination. Our recent paper demonstrated the potential of correlating EEM spectra with cell viability: A375 cells were exposed to UV radiation as a physical stress factor, resulting in a decrease of viability up to ca. 20%, confirmed by the standard MTT test. Their EEM spectra were processed by the unfolded partial least-squares (UPLS) regression, and further statistical evaluation revealed high accordance between the two methods of cell viability testing in terms of accuracy, precision, and correlation.^23^

However, it should be emphasized that our research and the literature data presented above concern only specific cell lines, specific factors/stimuli, and specific processes. Moreover, the nature of the changes recorded in the studied “fingerprints” (i.e., EEM, infrared, Raman spectra, electronic tongue signals) remains not fully explained in many cases. Hence, there is a need to explore the information hidden in the complex signals to reveal the possible mechanisms of the observable changes both for a better understanding of the studied processes and for higher reliability of the developed methods for cell culture monitoring. This article aims to fulfill these two goals—we employed the parallel factor analysis (PARAFAC) model to decompose excitation–emission matrices of two adherent skin cell lines, providing excitation and emission profiles (loadings) and relative concentrations (scores) of fluorophores present in the investigated cell cultures. The obtained PARAFAC scores are very helpful, both to explain which fluorophores can be regarded as correlating with the cell viability changes and to establish a reliable regression model for very accurate and precise determination of viability in A375 and HaCaT cell cultures treated with oxaliplatin.

## Experimental Section

### Reagents and Materials

In our experiments, two adherent human skin cell lines were used: A375 (malignant melanoma) and HaCaT (keratinocytes). The cells were purchased from ATCC (Manassas, VA, USA) and Deutsches Krebsforschungszentrum Stabsstelle Technologietransfer (Heidelberg, Germany), respectively. DMEM high glucose, fetal bovine serum (FBS), penicillin, streptomycin, l-glutamine, and phosphate buffer saline (PBS) were purchased from Biowest (Nuaillé, France). Tryple Express was obtained from Gibco (Waltham, MA, USA). MTT salt (3-(4,5-dimethylthiazol-2-yl)-2,5-diphenyltetrazolium bromide) was supplied by Sigma-Merck (Poznań, Poland). Dimethyl sulfoxide (DMSO) was purchased from POCH (Gliwice, Poland). Oxaliplatin (OXA) was from Selleckchem (Munich, Germany).

The cell culture condition was analyzed using an inverted fluorescence microscope (Olympus, Center Valley, PA, USA). The absorbance measurements were performed using a multiwell plate reader, Cytation-3 (BioTek, Instruments, Inc., Winooski, VT, USA).

### Cell Cultures and Cell Viability Determination

A DMEM high glucose base supplemented with 10% FBS, 1% penicillin, streptomycin, and 1% l-glutamine was used to culture A375 and HaCaT cell lines. They were subcultured every 2 days. Briefly, cells were rinsed with 1 mL of PBS after medium removal, followed by their detachment from the surface of the culture vessel using 1 mL of Tryple Express. Then the cell suspension was centrifuged for 3 min using 2000 rpm. The appropriate cell density (1.5 × 10^5^ cells/mL) was obtained by suspending the pellet in the cell culture medium. Next, cells were seeded into a 96-well plate (100 μL per well). After 24 h, oxaliplatin solutions were added in the concentration range of 5–1000 μM. After 24 h of incubation of the cells with the compound, the medium was exchanged. The cell preparation protocol was used for the cell viability tests and fluorescence analysis.

The MTT assay was used to determine cell viability. First, 100 μL of MTT salt solution (0.5 mg/mL) was added after removing the medium from the wells. After 4 h of incubation, the solution was gently removed from the wells, and 100 μL of DMSO was added. Then, the absorbance of the solution was measured at a wavelength of 570 nm. The cell viability was calculated as the ratio of the test samples’ mean absorbance to the control samples’ mean absorbance (not exposed to the oxaliplatin).

### The Acquisition of EEM Fluorescence Data

Excitation–emission matrices of A375 and HaCaT cell lines treated with oxaliplatin were acquired by a Synergy Neo 2 Hybrid Multi-Mode Microplate Reader fluorescence spectrometer (BioTek Instruments, Inc., Winooski, VT, USA) using a Corning 96-well High Content Imaging Plate (Corning 4517, Corning, Inc., Corning, NY, USA). A hand-written measurement protocol in which subsequent emission spectra were recorded at decreasing excitation wavelengths from 500 to 250 nm was used for the EEM fluorescence data collection. The data interval in excitation mode was 10 nm. The range of the recorded emission spectra depended on the excitation wavelength at which the spectrum was acquired. For λ_ex_ in a range of 290–500 nm, the emission was recorded from λ_em_ = λ_ex_ + 20 nm to 650 nm, whereas for λ_ex_ < 290 nm, the emission was measured in the range of 300–650 nm. The resolution of all emission spectra was 5 nm. All fluorescence measurements were performed at 37 °C, using bottom optics. Each sample, treated with oxaliplatin at various concentration levels, was measured in 12 replicates.

### Data Analysis

The fluorescence measurements of each sample were described by EEM of dimensions 71 × 26 (λ_em_ × λ_ex_). Parallel factor analysis (PARAFAC) was used as a multiway chemometric method to decompose EEMs and predict the cell viability reference. Since the total number of samples was 144 (2 cell lines ×6 concentrations of administered oxaliplatin ×12 replicates), the multiway array subjected to PARAFAC modeling was of size 144 × 71 × 26 (samples × λ_em_ × λ_ex_). The chemometric analysis was performed in Solo version 8.9 (Eigenvector Research Inc., Manson, US), while the figures were generated with Origin (OriginLab Corporation, Northampton, MA, USA) or MS Excel (Microsoft, Redmond, US) software.

## Results and Discussion

### The Influence of Oxaliplatin on Cell Cultures

The presented studies were focused on the effect of oxaliplatin on the viability of skin cells: normal HaCaT and tumor A375. Oxaliplatin is a widely used anticancer drug. It interacts with the cell’s nucleic acid, creating cross-links with DNA, destabilizing the DNA structure, and resulting in its breakage. That leads to the inhibition of fundamental biological processes and cell death. As a part of the experiment, the viability and morphology of normal and cancer skin cells were monitored after incubation with oxaliplatin. The cells were incubated with the compound for 24 h, and various concentration ranges were used: A375–5, 50, 250, 500, 1000 μM and HaCaT–5, 25, 100, 250, 500 μM. Oxaliplatin concentrations were selected to obtain the broadest possible cell viability range: from high values (approximately 90%) to low values (approximately 10%). The cells were microscopically observed to assess their morphology changes, and the MTT cell viability test was performed.

**Figure 1:**
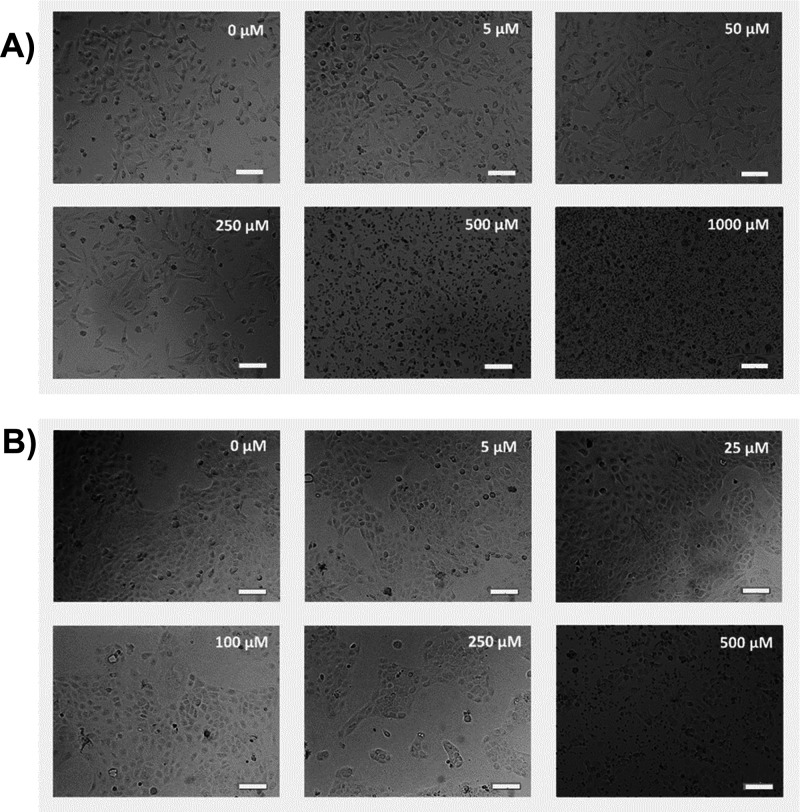
Bright field microscopic pictures of cells after 24 h of incubation with oxaliplatin: A375 cell line (A), HaCaT cell line (B). Scale bar 100 μm.

Figure 1A presents the microscopic pictures of A375 cells after incubation with an oxaliplatin solution. The effect of compound concentration on cell density and morphology can be seen. After incubating the tumor cells with 5 and 50 μM oxaliplatin, no changes in cell morphology were noticed compared to untreated control samples. On the other hand, after incubation of A375 cells with OXA at a concentration of 250 μM, a slight decrease in the cell density in the culture well was noticed. That could be due to the detachment of dead cells. When tumor cells were incubated with oxaliplatin at concentrations of 500 and 1000 μM, significant cell fragmentation and disintegration were noted. The administered concentration of oxaliplatin led to cell death and breakdown, as shown in the pictures. Figure 1B presents HaCaT cells after 24 h of incubation with various concentrations of oxaliplatin. As can be seen, as the concentration of OXA increases, the cell density decreases, and the cell morphology changes. In the case of normal cells, no changes in the cell morphology were noticed after incubation with 5 and 25 μM oxaliplatin compared to control cells. Incubation with the compound at a concentration of 100 and 250 μM resulted in a slight decrease in cell density, while a concentration of 500 μM administered to the cells led to their disintegration.

**Figure 2:**
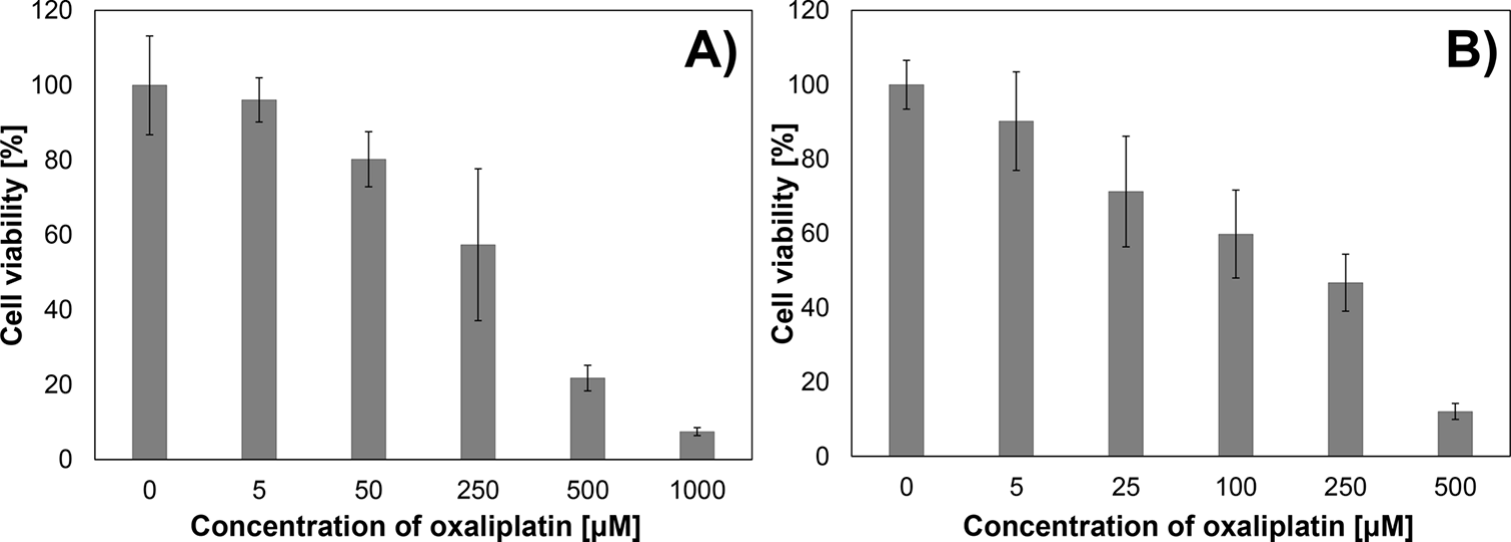
Viability of cells determined by the MTT test (mean ± SD, *n* = 5) after 24 h of incubation with oxaliplatin: A375 cell line (A), HaCaT cell line (B).

The graphs in Figure 2 show the dependence of the cell viability on oxaliplatin concentration. Both for cancer (Figure 2A) and normal (Figure 2B) cells, the cell viability decreases with increasing OXA concentration. In the case of A375, the viability of cells after incubation with the compound at 5 and 50 μM concentrations was high–approximately 90 and 80%, respectively. Incubation of the tumor cells with OXA at a concentration of 250 μM decreased cell viability to a level of approximately 60%. At 500 and 1000 μM concentrations of oxaliplatin, the viability of A375 cells was very low—approximately 10%. The viability of HaCaT cells after incubation with oxaliplatin is presented in Figure 2B. In this case, the incubation with the compound at a concentration of 5 μM caused a slight decrease in the cell viability—to a level of approximately 90%. After incubating the cells with 25 and 100 μM oxaliplatin, the viability of the cells decreased to a level of 60–70%. On the other hand, after incubation with OXA at a concentration of 250 μM, the viability of the HaCaT cells was reduced by about half. Very low cell viability (around 10%) was observed after incubation with a compound concentration of 500 μM. The MTT viability test results are consistent with microscopic observations. The obtained results clearly show the cell viability’s dependence on the concentration of administered oxaliplatin.

### Excitation–Emission Matrices of Oxaliplatin-Treated Cell Cultures

**Figure 3:**
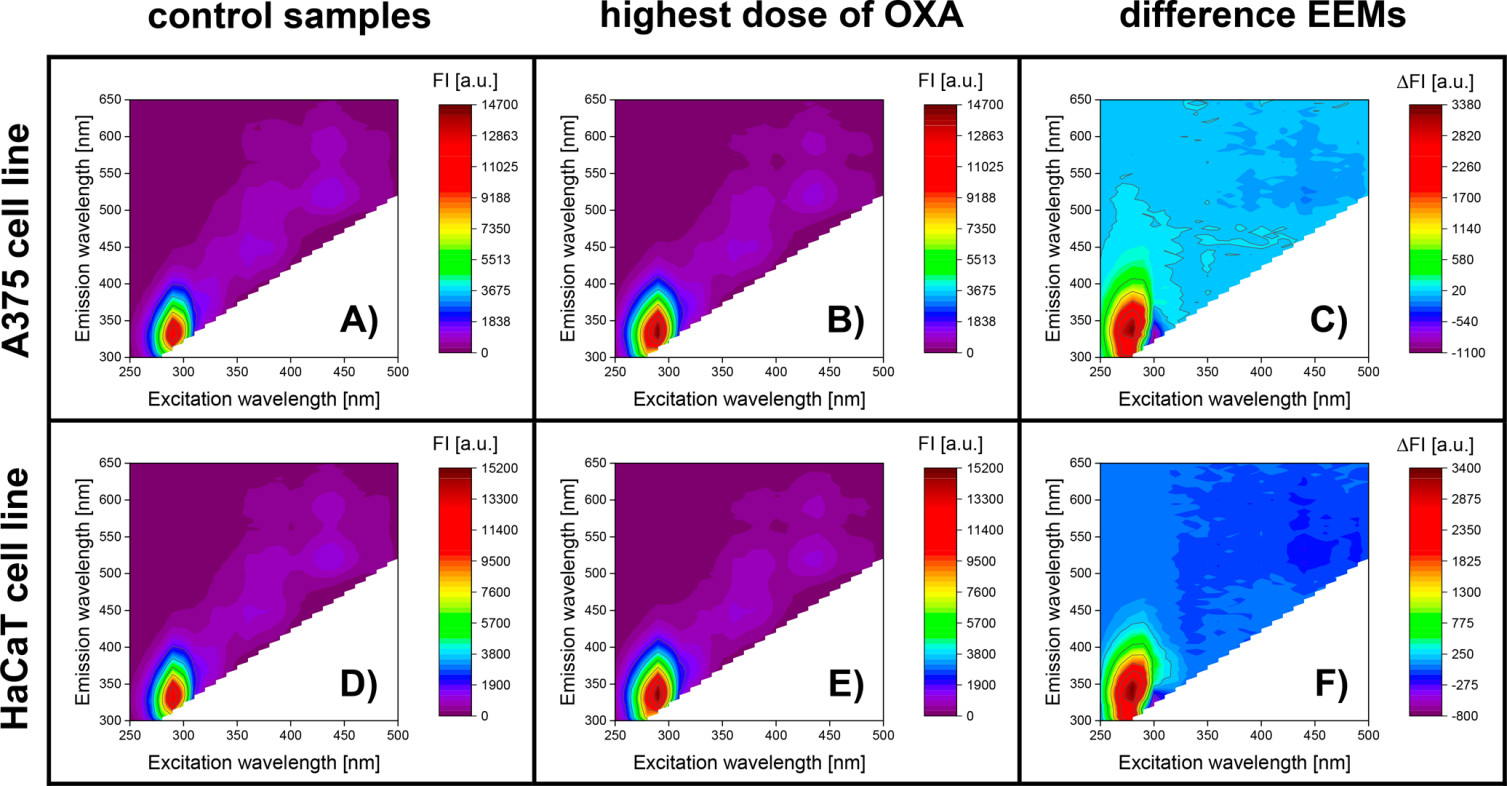
Influence of oxaliplatin on the excitation–emission matrices (EEMs) of A375 (A–C) and HaCaT cell line (D–F): EEMs of cell cultures not exposed to oxaliplatin (A, D), EEMs of cell cultures incubated with the highest dose of oxaliplatin (B, E), EEM difference spectra of cell cultures exposed to the highest dose of oxaliplatin (C, F). The difference spectra were obtained by subtracting the EEM spectrum of the cell culture not subjected to the oxaliplatin from the EEM spectrum of the cell culture treated with the highest dose of the drug (i.e., C = B – A; F = E – D).

The influence of oxaliplatin on the fluorescent landscapes (EEMs) of A375 and HaCaT-adherent cell lines is indicated in Figure 3. First of all, it can be seen that control samples of both cell lines under investigation exhibit fluorescence in the same spectral regions (Figure 3A,D). Thus, the cell cultures not treated with oxaliplatin fluoresced very strongly around 290 nm/340 nm (λ_ex_/λ_em_), while signals of lower intensity can also be distinguished around 360 nm/440 nm, 390 nm/500 nm, 390 nm/590 nm, as well as 430 nm/520 nm and 430 nm/590 nm. It should be mentioned that these results are consistent with our previous work,^23^ and the observed fluorescent signals can be related not only to endogenous fluorophores present in the cells (e.g., amino acids, proteins, cofactors, vitamins, lipids, porphyrins^24,25^) but also to the fluorescent components of the cell culture medium, such as amino acids and vitamins.^26,27^ To show which spectral ranges were most affected by the action of oxaliplatin, excitation–emission matrices of A375 and HaCaT cell lines after 24 h of incubation with the highest concentrations of the drug are presented in Figure 3B,E, respectively. Additionally, the difference EEM spectra obtained by subtracting controls from samples exposed to the highest concentration of the stress factor are also included in Figure 3C,F. Based on the analysis of the presented spectra, one can see that the exposure of A375 and HaCaT cells to oxaliplatin results in the most significant alternations of the fluorescence signals around λ_ex_ ∈ (250 nm, 300 nm), λ_em_ ∈ (300 nm, 450 nm), which might correspond to the changes of the content of such endogenous fluorophores as aromatic amino acids.^24,25,28^ Moreover, the difference spectra reveal that in the mentioned spectral range, a substantial increase in the fluorescence can be observed, and its decrease at approximately 290 nm/350 nm, which suggests the presence of at least two fluorophores (Figure 3C,F). No significant changes in the spectral regions most typically attributed to the content of cofactors and vitamins^25,28^ were observed between control samples and samples of cell cultures affected by the highest concentration of oxaliplatin (Figure 3A,B and D,E) compared to the previously reported results.^23^ On the other hand, based on the difference EEM spectra, it can be seen that fluorescence intensity in the ranges of λ_ex_ ∈ (300 nm, 340 nm), λ_em_ ∈ (360 nm, 460 nm), and λ_ex_ ∈ (340 nm, 430 nm), λ_em_ ∈ (420 nm, 480 nm) slightly decreases under the influence of the highest concentration of oxaliplatin. This phenomenon might be related to an inner filter effect, which may be due to the fluorophores’ high concentration and the samples’ compositional complexity.^29,30^ Nevertheless, the interaction of oxaliplatin affects the excitation–emission matrices of both A375 and HaCaT cell lines, indicating that the cell viability’s decrease directly impacts the obtained fluorescence landscape. Due to the measurement protocol for fluorescent data acquisition used in this study (see the Acquisition of EEM Fluorescence Data section), fluorescence intensity was always measured at emission wavelengths higher than the excitation wavelengths. Thus, Rayleigh scattering was not observed in our data. In addition, no evidence of Raman signals in the obtained excitation–emission matrices was noted (Figure 3).

## PARAFAC Modeling of EEM Fluorescent Data of Cell Cultures

The application of excitation–emission matrix fluorescence spectroscopy for analyzing complex samples containing biogenic fluorophores can allow their simultaneous detection in qualitative and quantitative analysis.^26^ In addition, this multiwavelength technique can also be used when the presence and change in the concentration of fluorophores contained in the sample can be directly or indirectly correlated with the parameters of biotechnological processes and cell culture, e.g., in the pattern-based sensing approach.^22−24,28^ Due to the complexity of the obtained EEM spectra, it is necessary to use multivariate analysis methods that enable the extraction of the most relevant spectral information.^31^

Our previous work^23^ showed that the information encoded in the excitation–emission matrices of a UV-treated cell culture could be correlated with the viability of the cells determined with the MTT test by utilizing the unfolded partial least-squares (UPLS) regression. Although UPLS is often used for second-order data calibration,^32−34^ the application of chemometric methods dedicated to the processing of multiway data (e.g., EEMs) can result in more adequate, robust, and chemically interpretable models.^35^ Therefore, in the framework of the presented study, we employed parallel factor analysis (PARAFAC) to decompose the excitation–emission matrices of two skin cell lines (A375–malignant melanoma, HaCaT–keratinocytes). The PARAFAC model is one of the most widely used chemometric methods for multiway fluorescence data analysis.^36^ Among the main advantages of PARAFAC modeling is its uniqueness, which means it allows for the decomposition of EEM spectra into trilinear components, which can provide estimates of the actual excitation and emission spectra (loadings) of fluorophores present in the analyzed samples, as well as their relative concentrations (scores). However, this feature can only be achieved when the data behave according to Beer’s law and the correct number of components is chosen.^35,36^ The basic strategies used to validate the PARAFAC model include split-half experiments, core consistency, investigation of residuals, and assessing the physical reasonability of the obtained spectral loadings in relation to the previously possessed chemical knowledge about the experiment. A detailed description of PARAFAC validation can be found elsewhere.^35^

Before the PARAFAC modeling of fluorescent data of cell cultures, a large amount of missing data in the EEM spectra resulting from the applied procedure for data acquisition (the white area on the EEMs, Figure 3) was dealt with to stabilize the decomposition of the spectra. For this purpose, the regions of the EEM spectra where first-order Rayleigh scattering occurs (λ_ex_ = λ_em_) were interpolated, whereas zeros were inserted in the area below.^37^ Next, eight PARAFAC models were built using different numbers of components (1–8). Since the previous analysis of the raw excitation–emission matrices of cell cultures suggested that at least five components should be expected for appropriate PARAFAC modeling (see the Excitation–Emission Matrices of Oxaliplatin-Treated Cell Cultures section), we decided to closely evaluate models where EEM cell culture data are described by five to eight components. Non-negativity was imposed on all model parameters. The investigation of the residuals for those models showed that not much extra information was added by increasing the number of components beyond five, indicating that the five-component model was modeling the data well. Finally, we applied a split-half validation, which relies on dividing data into two parts and independently fitting PARAFAC models on both parts. The idea behind this validation method is that if the correct number of components is chosen, the obtained models should have the same excitation and emission loadings.^35,36^ In our case, we split the data so that one set consisted of EEMs of only the HaCaT cell line and the other split consisted of fluorescent data of the A375 cell line. Emission and excitation loadings for five- and six-component PARAFAC models estimated during the validation procedure are depicted in Figure S1 (Supporting Information). First of all, it can be easily seen that the obtained spectral profiles of five-component models are chemically meaningful and characterized by a very similar shape and peak position in each investigated case (Figure S1A,B). On the other hand, when the sixth component was introduced during PARAFAC modeling (Figure S1C,D), changes in the shape of the signal, as well as spectral shifts for the third (blue line) and the fifth (orange line) components, were noted. Moreover, the greatest changes in the position and shape of the spectral loadings, both in the emission and excitation modes, are seen for the sixth component (gray line), which means that such a number of components might be too high to describe the EEM data of cell cultures adequately. Therefore, it was concluded that a five-component model best describes the fluorescence data of A375 and HaCaT cell cultures.

**Figure 4:**
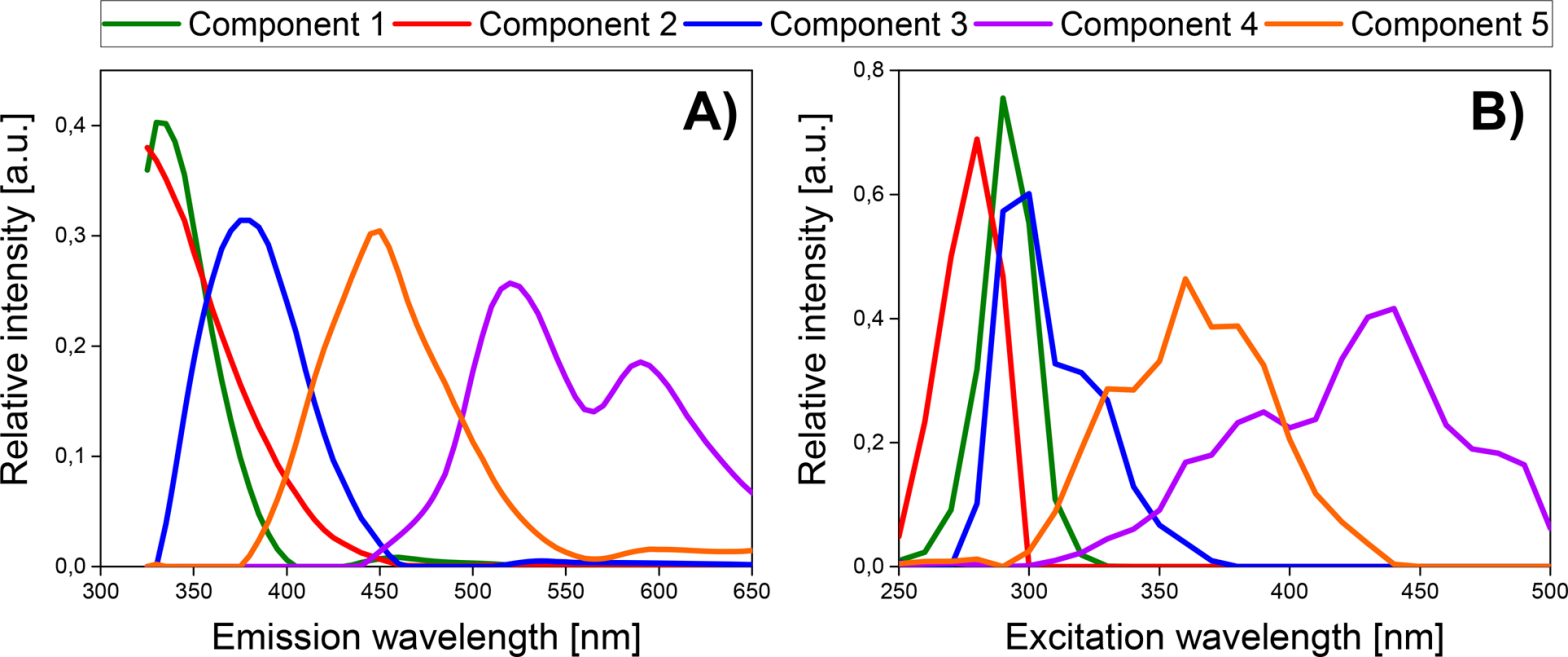
Emission (A) and excitation (B) mode loadings from the five-component PARAFAC model of fluorescence data of A375 and HaCaT cell cultures treated with oxaliplatin.

The five-component PARAFAC model of A375 and HaCaT cell cultures affected by the action of oxaliplatin explained 99.93% of the variance of EEM fluorescence data. The excitation and emission loadings obtained during the decomposition of excitation–emission matrices by the five-component PARAFAC model are shown in Figure 4. These designated spectral profiles represent the underlying pure spectra of fluorophores present in A375 and HaCaT cell cultures. The spectral position of the first and second components indicates that they are most likely aromatic amino acids, which might form structural proteins,^25^ but are also components of the cell culture medium.^26^ The pair of excitation/emission wavelengths of the first component was 290 nm/330 nm, which, under cell culture conditions, might correspond to tryptophan.^24^ At the same time, the second component is most likely tyrosine, which fluoresces at 275 nm/300 nm.^25^ In our case, the excitation maxima of the second component were at 280 nm, while due to the exclusion of the emission wavelength in the range of 300–320 nm, it is difficult to indicate the exact position of the emission maximum. Notwithstanding, the obtained emission profile suggests that the maximum emission of the second component is below 325 nm (Figure 4). The third component is characterized by excitation and emission maxima at 300 and 380 nm, respectively. Therefore, it appears that this component can be related to pyridoxine.^24,28^ Interestingly, the fourth component characterizes a bimodal emission profile, with maxima at 520 and 590 nm. This shape of emission spectra most often describes endogenous porphyrins.^25^ However, the position of the emission maxima is rather unusual for this group of fluorophores. The analysis of the corresponding excitation profile suggests that two maxima in this mode are present, at approximately 390 and 440 nm, which might indicate the presence of flavins.^25^ However, it is also possible that the fourth component is a composite of two fluorophores,^26^ one of which may be riboflavin, usually fluorescent around 365 nm/520 nm. The excitation/emission maxima positioned at 360 nm/450 nm belonged to the fifth component, which may correspond to coenzymes, e.g., NADH.^26^

**Figure 5:**
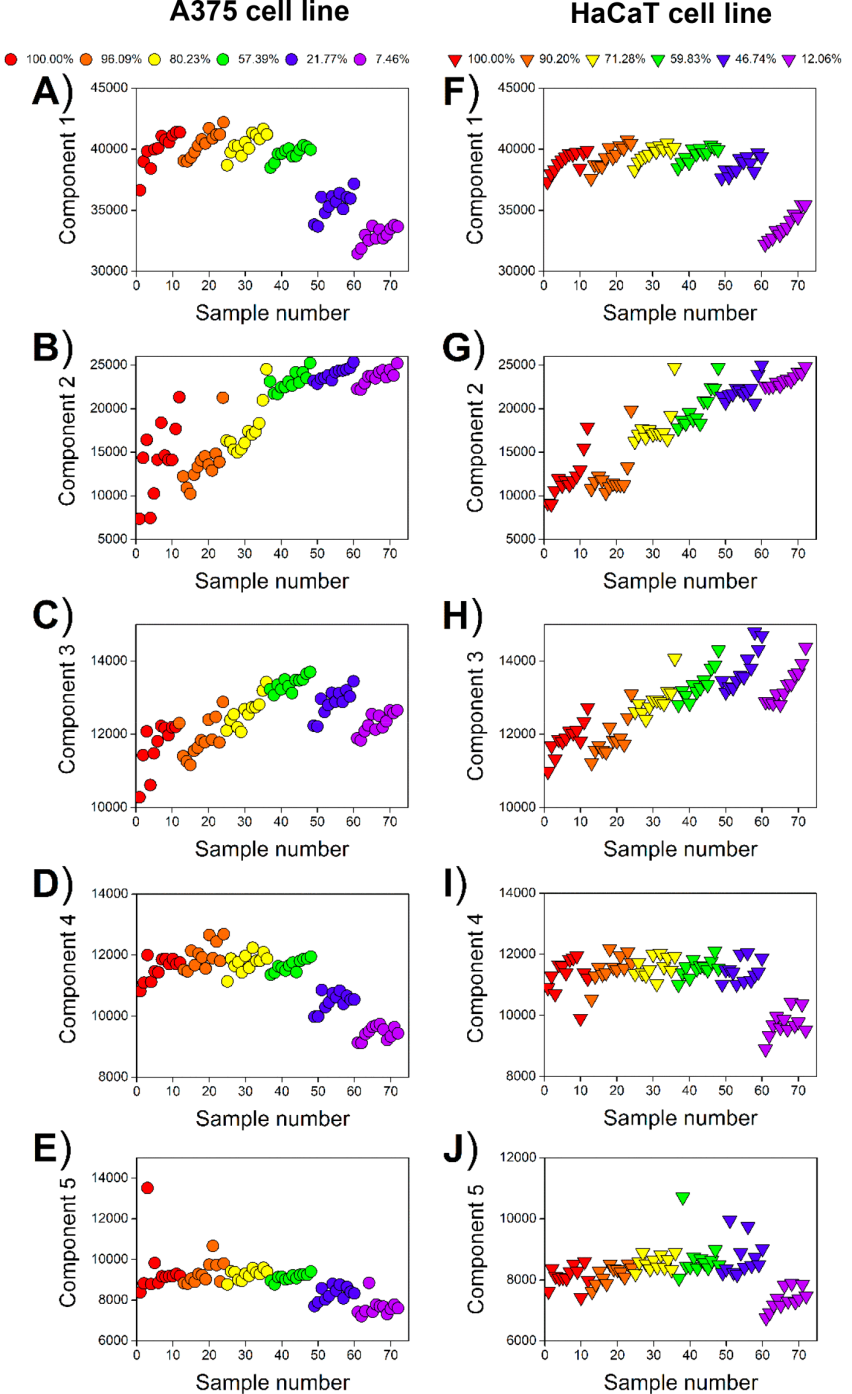
Score plots from the five-component PARAFAC model of fluorescence data of A375 (A–E) and HaCaT (F–J) cell cultures: component 1 (A, F); component 2 (B, G); component 3 (C, H); component 4 (D, I); component 5 (E, J). The values inserted in the legend correspond to the mean cell viability determined by the MTT test.

The relative concentrations (scores) of fluorophores present in samples of A375 and HaCaT cell cultures are depicted in Figure 5. As can be seen, the decrease in cell viability is well reflected by the changes in the score values for all five PARAFAC components. It should be underlined that scores on components 1–5 change similarly in the case of both investigated cell lines. As cell viability decreases, the relative concentration of components 1, 4, and 5 decreases (Figure 5A,F, D,I, and E,J), while for components 2 and 3, an increase in the score values is noted (Figure 5B,D and C,H). Interestingly, in the case of the A375 cell line, the relative concentration of component 3 slightly decreases for samples with lower viability (Figure 5C), which may be related to the inner filter effect. It is worth mentioning that for components 1–3 a slight systematic drift in score values was observed; however, this effect needs further investigation to explain its mechanism.

Viability tests are usually not applied to observe only one value of viability at a respective drug dose but rather to trace the trend observed when the magnitude of this stress factor increases (e.g., dose-dependent effect in drug screening). Therefore, the scores obtained by PARAFAC were subjected to statistical verification of their accordance with the results of the MTT tests. The statistical analysis was focused on detecting significant differences between the same sample groups (cells exposed to various doses of oxaliplatin). First of all, a one-way ANOVA was calculated for the viability MTT test results for both cell lines. The obtained F values suggested that one or more considered samples differed significantly (Table 1 and Table 2 for A375 and HaCaT cells, respectively). Results of the Tukey posthoc test showed insignificant differences (*p* > 0.05, red-marked) only for cells exposed to the following doses of oxaliplatin (μM): 0 vs 5, 0 vs 50, 5 vs 50, 500 vs 1000 (for A375 cells) and 0 vs 5, 5 vs 25, 25 vs 100, 100 vs 250 (for HaCaT cells). All other groups were discernible from each other, at least at a 5% confidence level (*p* ≤ 0.05, green-marked). A significant difference from the control (“0” dose) was observed for various amounts of oxaliplatin in the studied cell lines–at 250 μM for A375 cells and at 25 μM for HaCaT cells. All these observations serve as a reference for further studies—EEM coupled with PARAFAC should provide at least the same discrimination; i.e., green marks should appear at least wherever they were observed in the case of the MTT test.

**Table 1:**
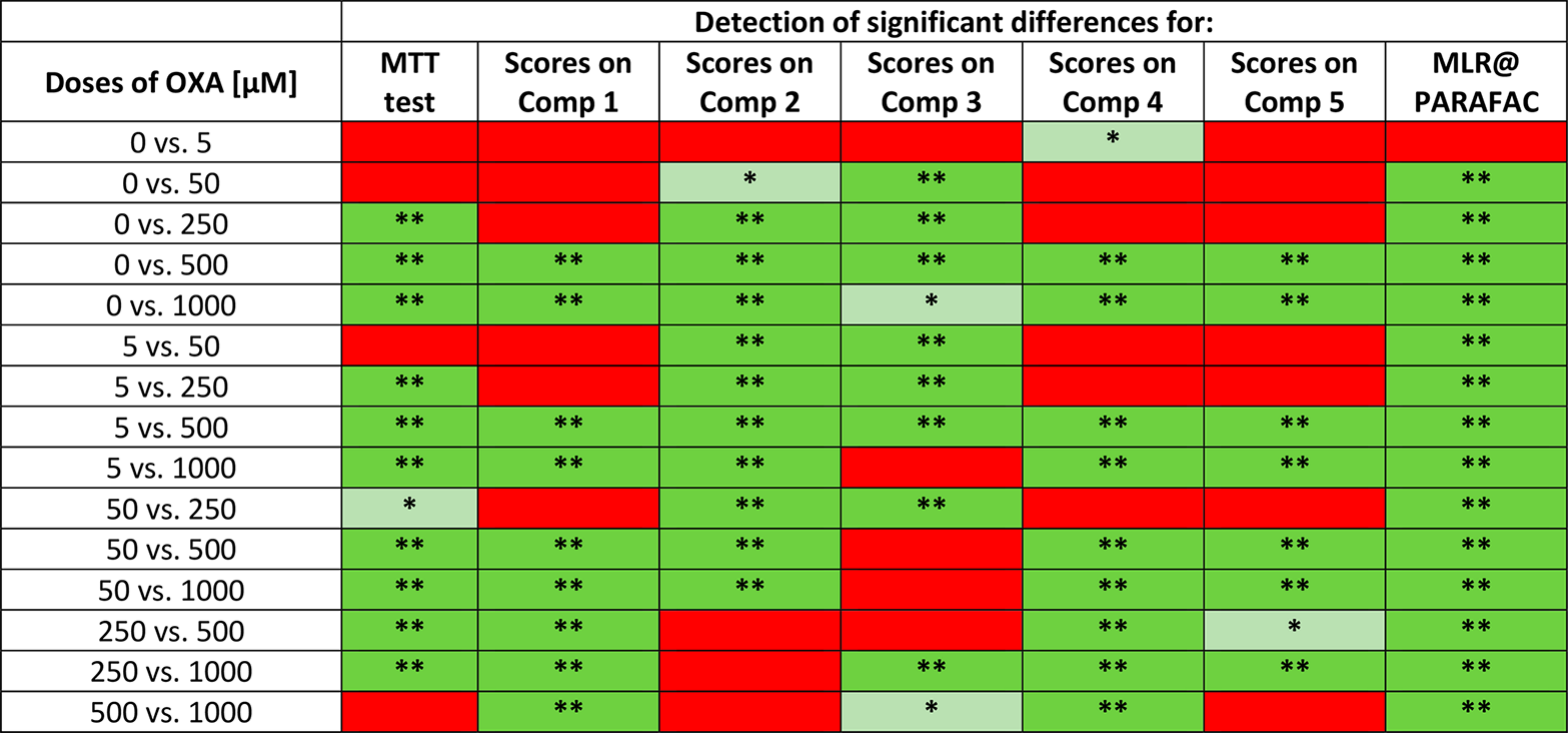
Detection of Significant Differences between the Viabilities of A375 Cells Observed for Various Doses of Oxaliplatin, Determined by the Standard MTT Test and Based on PARAFAC Scores^a^

a The results based on Tukey post-hoc test showed significant (*p* ≤ 0.05, green-marked) and insignificant (*p* > 0.05, red-marked) differences. *p < 0.05; **p < 0.01; MLR@PARAFAC–MLR based on PARAFAC scores of components 1–5.

**Table 2:**
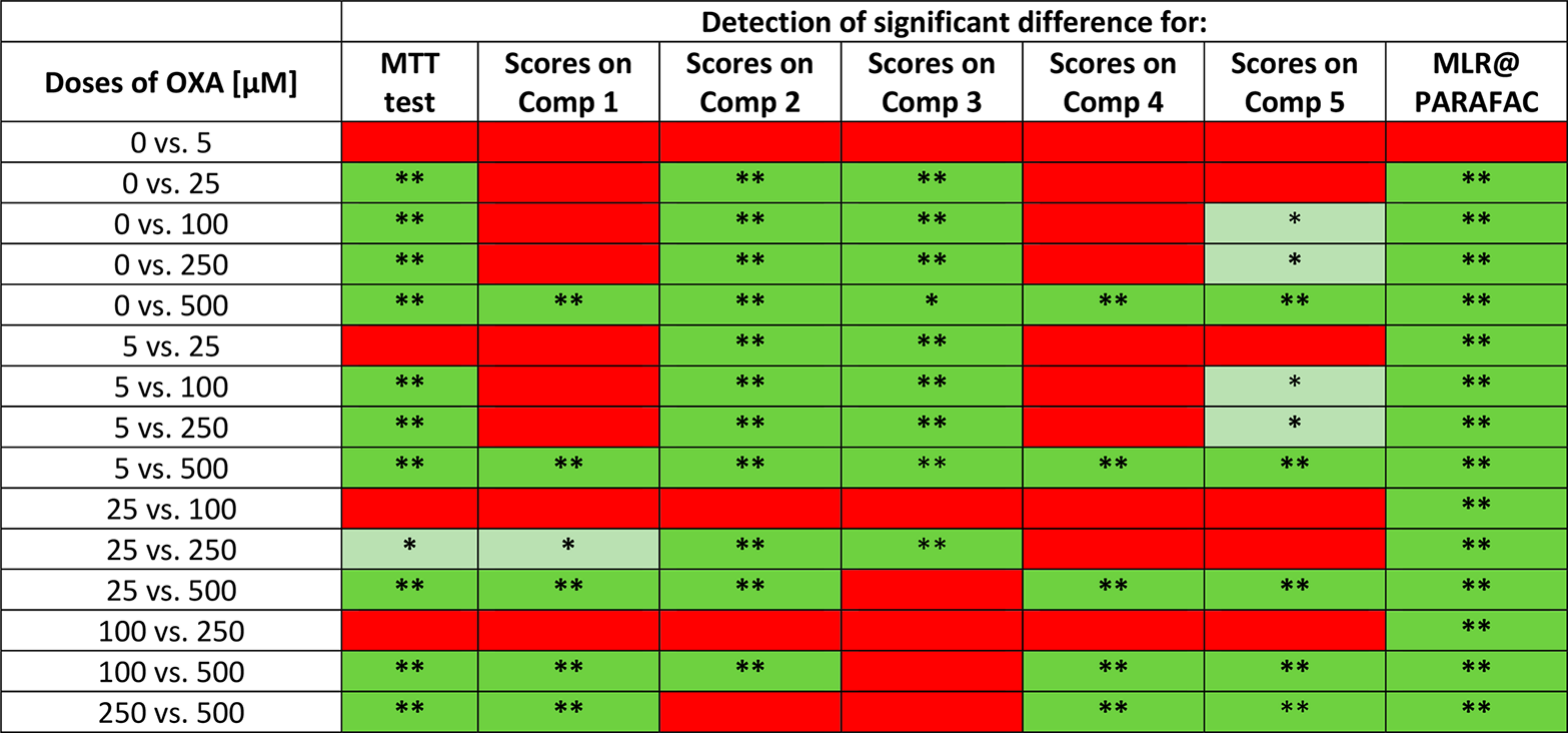
Detection of Significant Differences between the Viabilities of HaCaT Cells Observed for Various Doses of Oxaliplatin, Determined by the Standard MTT Test and Based on the PARAFAC Scores^a^

a Results based on Tukey post-hoc test showed significant (*p* ≤ 0.05, green-marked) and insignificant (*p* > 0.05, red-marked) differences. *p < 0.05; **p < 0.01; MLR@PARAFAC–MLR based on PARAFAC scores of components 1–5.

Scores obtained by PARAFAC, when considered one by one, could only partially reflect the MTT test results. For example, the significant difference between “0” and “250” doses in A375 cells (Table 1) detected by the MTT test was not stated based on components 1, 4, and 5. On the other hand, components 2 and 3 did not discern “250” and “500” doses, whereas utilizing the MTT test was possible. In the case of HaCaT cells, component 2 exhibited the greatest ability to distinguish the samples exposed to various doses of oxaliplatin; however, it failed in the “250 vs 500” case, where the MTT test was superior. It is worth noting that the results of the posthoc Tukey test for PARAFAC components 1–5, visible in Tables 1 and 2, provide statistical verification of significant differences between sample groups, in good agreement with the patterns of the PARAFAC scores presented in Figure 5.

**Figure 6:**
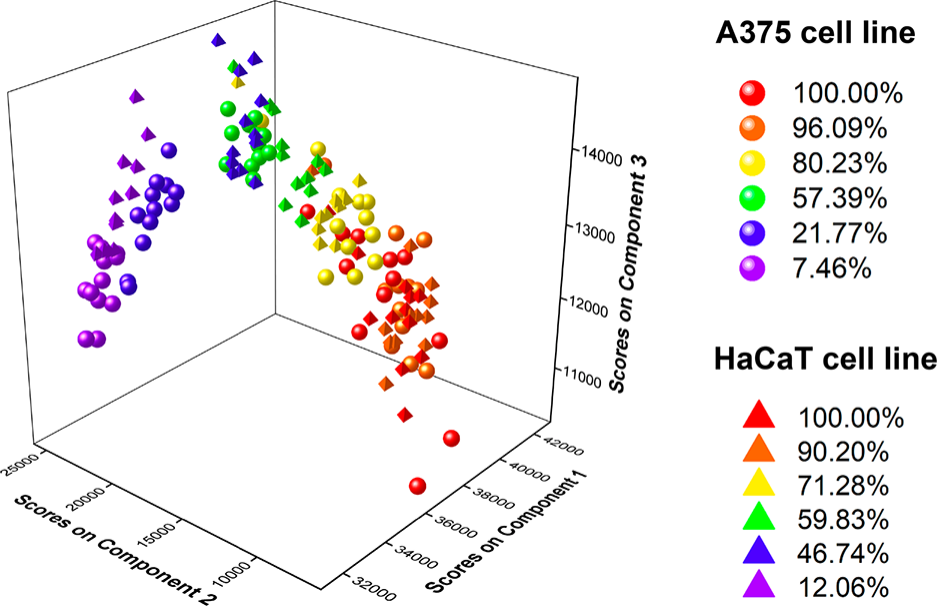
PARAFAC model score plot for predicting cell viability in oxaliplatin-treated A375 and HaCaT cell cultures. The values inserted in the legend correspond to the mean cell viability determined by the MTT test.

The analysis of the relative concentration of fluorophores indicates that the most significant changes that may be associated with the MTT test cell viability results are observed for the first three components. Accordingly, the relative concentrations of these components were plotted on a three-dimensional plot to show the separation between cell culture samples of different viabilities (Figure 6). As would be expected, the points corresponding to the different viability levels are grouped according to the reference values obtained with the MTT test for both cell lines. It is worth noting that the samples with higher viability (exposed to oxaliplatin at 0, 5 μM – A375; 0, 5, 25 μM – HaCaT) overlap to some extent. However, this overlap reflects that cell viability does not change significantly for those samples, as evidenced by the MTT test results and microscopic observations. That confirms that the changes in the relative concentration of fluorophores in cell cultures may indeed be associated with cell viability, and EEM fluorescence spectroscopy is an appropriate technique for viability studies. Thus, the developed PARAFAC model can be utilized for the viability prediction of both A375 and HaCaT cell lines.

### Viability Prediction Based on PARAFAC Scores

**Figure 7:**
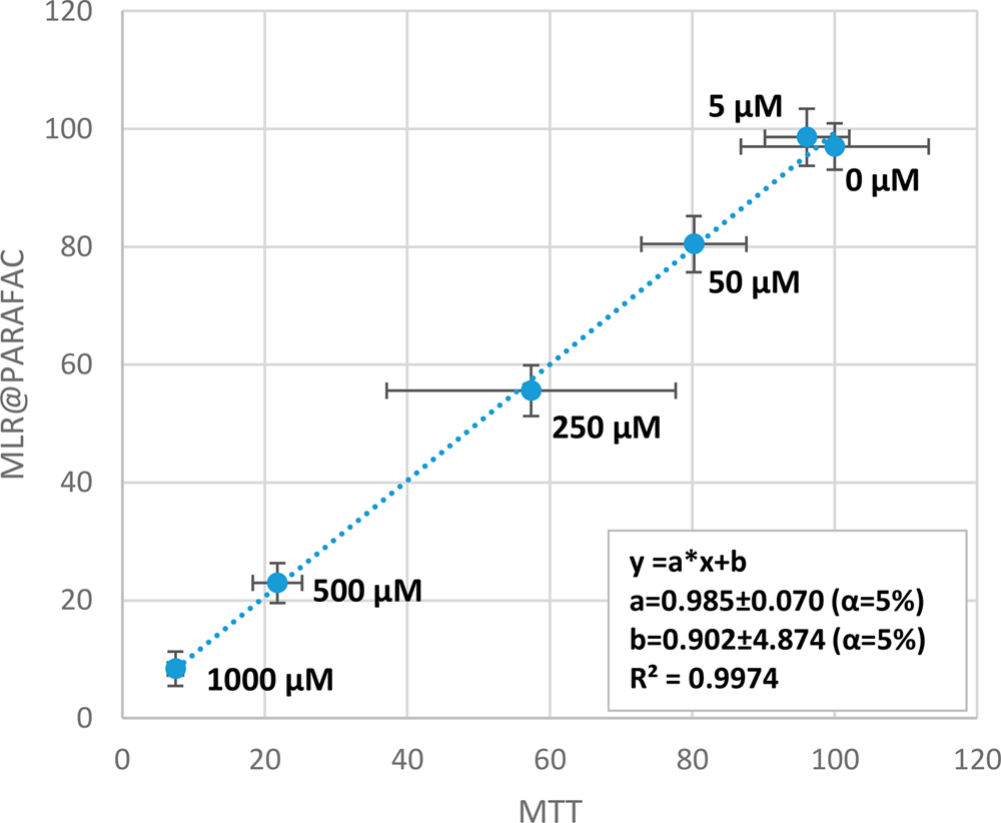
Correlation of A375 cell viability results obtained with the use of MTT tests and EEM fluorescence combined with MLR based on PARAFAC scores of components 1–5. Doses of oxaliplatin are given for each point representing mean ± SD. Linear fit revealed that the confidence intervals for “*a*” and “*b*” at α = 5% include the ideal values for such a comparison (*y* = *x*, thus *a* = 1, *b* = 0), which indicates that the differences between the mean results of the MTT test and EEM fluorescence combined with MLR@PARAFAC are only due to random errors (do not have statistical significance).

**Figure 8:**
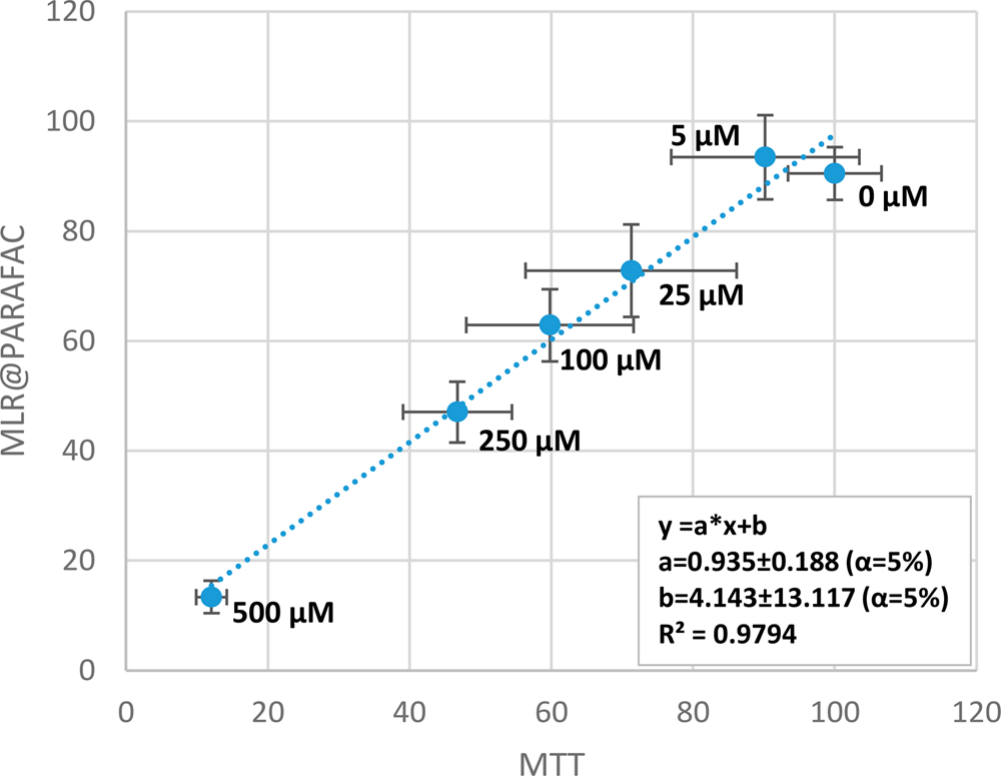
Correlation of HaCaT cell viability results obtained with the use of MTT tests and EEM fluorescence combined with MLR based on PARAFAC scores of components 1–5. Doses of oxaliplatin are given for each point representing mean ± SD. The linear fit revealed that the confidence intervals for “*a*” and “*b*” at α = 5% include the ideal values for such a comparison (*y* = *x*, thus *a* = 1, *b* = 0), which indicate that the differences between the mean results of the MTT test and EEM fluorescence combined with MLR@PARAFAC are only due to random errors (do not have statistical significance).

As in the case of both cell lines, the complementary importance of the individual PARAFAC components was noted, so we combined the information gained by all of them and established multiple linear regression (MLR) models for predicting cell viability. As inputs, scores on components 1–5 were applied in the case of both cell lines. The cross-validation results provided good accordance with the MTT data (Figures S2 and S3 in the Supporting Information). The correlation between the results obtained by the MTT test and MLR coupled with PARAFAC was verified and is presented in Figures 7 and 8 for A375 and HaCaT cells, respectively. Very high determination coefficients were observed: *R*^2^ = 0.997 for A375 cells and *R*^2^ = 0.979 for HaCaT cells, which indicates that only ∼0.3% (A375) or ∼2.1% (HaCaT) of variability in this data set is not explained by the linear fit—random errors can easily explain this very low value. In perfect accordance with the results, the ideal linear fit should be obtained between the results of the two studied methods, which can be expressed with the equation *y* = *x* (*y* = 1·*x* + 0). In our case, the values of slope (“*a*”) and bias (“*b*”) were close to the ideal values 1 and 0, respectively, for both cell lines. Moreover, their confidence intervals obtained for a confidence level of 95% (α = 5%) revealed that they contained the values of a perfect fit. In other words, *a* = 0.99 ± 0.07 does not significantly differ statistically from the ideal 1, and *b* = 0.90 ± 4.87 from 0 at α = 5% (A375 cells, Figure 7). The same can be concluded for HaCaT cells (Figure 8). This is another argument showing great accordance between the results obtained using MTT tests and the EEM coupled with chemometric methods.

The results obtained by MLR using all PARAFAC component scores were finally subjected to verification of their ability to discern cells exposed to various doses of oxaliplatin precisely in the same manner as presented above for separately considered PARAFAC components. Again, combining all components provided superior results compared to using single components. All samples exposed to various doses of oxaliplatin, except one, were discernible—all differences that were statistically significant and marked with green can be observed in the last column of Tables 1 and 2. Insignificant difference (red) was stated only in the case of the control (0 μM) and the smallest dose of the drug (5 μM) for both cell lines. However, such “0 vs 5” discrimination was also not achieved by the MTT test in both cell lines, so it can be concluded that the smallest dose does not change their viability. Moreover, MLR coupled with PARAFAC could distinguish samples indiscernible by the MTT test: 0 vs 50, 5 vs 50, 500 vs 1000 in the case of A375 cells, and 5 vs 25, 25 vs 100, 100 vs 250 in the case of HaCaT cells. It means that the presented approach is even more sensitive to viability changes than the MTT test; it can capture some subtle but detectable decreases in cell numbers. The same trend was observed previously in the case of cells treated by UV radiation as a stress factor modulating viability^23^ and is probably linked with the higher precision of the developed EEM fluorescence method.

## Conclusions

Ideally, cell viability assays should be safe and rapid, enabling reliable and efficient analysis. Additional advantages involve time- and cost-effectiveness as well as no interference with drugs or other stress factors to which the cell culture is subjected.^1^ Although dye exclusion assays (e.g., trypan blue stain assay), colorimetric assays (e.g., MTT test), fluorometric assays (e.g., Alamar Blue assay), luminometric assays (ATP assay), and flow cytometry methods, which are stated as “gold standards” in viability testing, usually fulfill these assumptions, several disadvantages must also be noted. They are time-consuming and labor-intensive for a large number of samples, sometimes suitable only for cell suspensions; various counting errors are possible (due to poor cell dispersion, incorrect dilution, contamination, air bubbles, etc.); different additional steps in the analysis can be essential (e.g., trypsinization of cell monolayers, formazan dissolution in the MTT assay). Moreover, such assays are also prone to interfere with the cell culture medium. They can be affected by incubation time, cell type and count, the presence of aggregates, and various environmental factors, including even light (MTT, XTT, LDH assays). Thus, it is postulated to perform more than one cell viability assay to obtain reliable results.^1^

Moreover, there is also a great need to observe viability changes in real-time, continuously, and nondestructively, especially in tissue engineering, spheroid cultures, and wherever sequences of various stimuli are fed to the culture. EEM fluorescence spectroscopy has the potential to meet all these requirements—as we showed in this paper, it enables obtaining accurate results with better sensitivity than the MTT test. Due to the use of PARAFAC, it is possible to study the mechanism of the observed viability changes, which can be directly linked to increasing/decreasing fluorophores in the cell culture medium. It must be emphasized, however, that the presented approach is not based on straightforward calibration because it does not utilize a specific interaction as standard viability assays; it relates subtle changes in EEM fluorescence spectra to a cell culture parameter. Therefore, one of the critical aspects of the analysis is to ensure that the obtained EEM spectra are not affected by factors influencing the fluorescence signal that is not due to cell culture conditions. Consequently, further work should focus on a thorough examination of the limitations of our method when various stress factors are applied. In addition, it requires optimization to shorten the analysis time. Therefore, additional work on these issues must be undertaken and is ongoing in our laboratory.

## Supporting Information Available

Split-half validation results for the five- and the six-component PARAFAC models (Figure S1); viability determination of A375 and HaCaT cells with MLR@PARAFAC (Figures S2 and S3) (PDF)
