# Supplementary material for: Excitation–Emission Matrix Fluorescence Spectroscopy Coupled with PARAFAC Modeling for Viability Prediction of Cells
Source: ACS Omega. 2023 Apr 27;8(18):15968–78. doi: 10.1021/acsomega.2c05383 (PMC10173342; doi:10.1021/acsomega.2c05383)
Supplement: Supplementary file 1 — ao2c05383_si_001.pdf [file ao2c05383_si_001.pdf]

Supporting Information

Excitation-emission matrix fluorescence spectroscopy coupled with PARAFAC modeling for viability prediction of cells

*Klaudia Głowacz[1], Sandra Skorupska[1], Ilona Grabowska-Jadach[1], Rasmus Bro[2], Patrycja Ciosek-Skibińska[1]**

[1] Chair of Medical Biotechnology, Faculty of Chemistry, Warsaw University of Technology, Noakowskiego 3, 00-664 Warsaw, Poland

[2] Department of Food Science, University of Copenhagen, Rolighedsvej 30, DK-1958, Frederiksberg C, Denmark

*Correspondence: patrycja.ciosek@pw.edu.pl

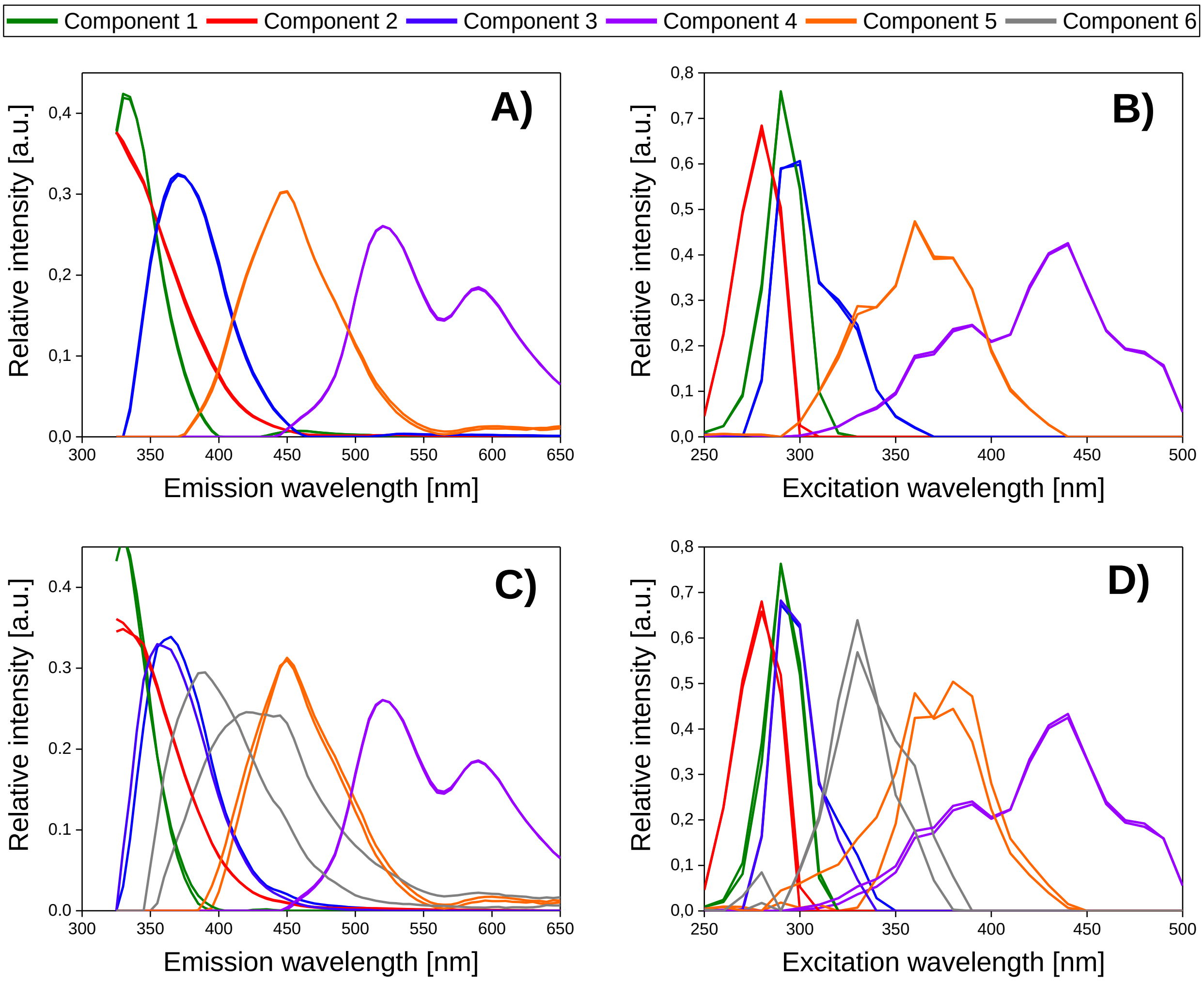

Figure S1. Emission (A, C) and excitation (B, D) mode loadings from five (A, B) and six (C, D) component PARAFAC models of the fluorescence data of A375 and HaCaT cell cultures derived during validation procedure.

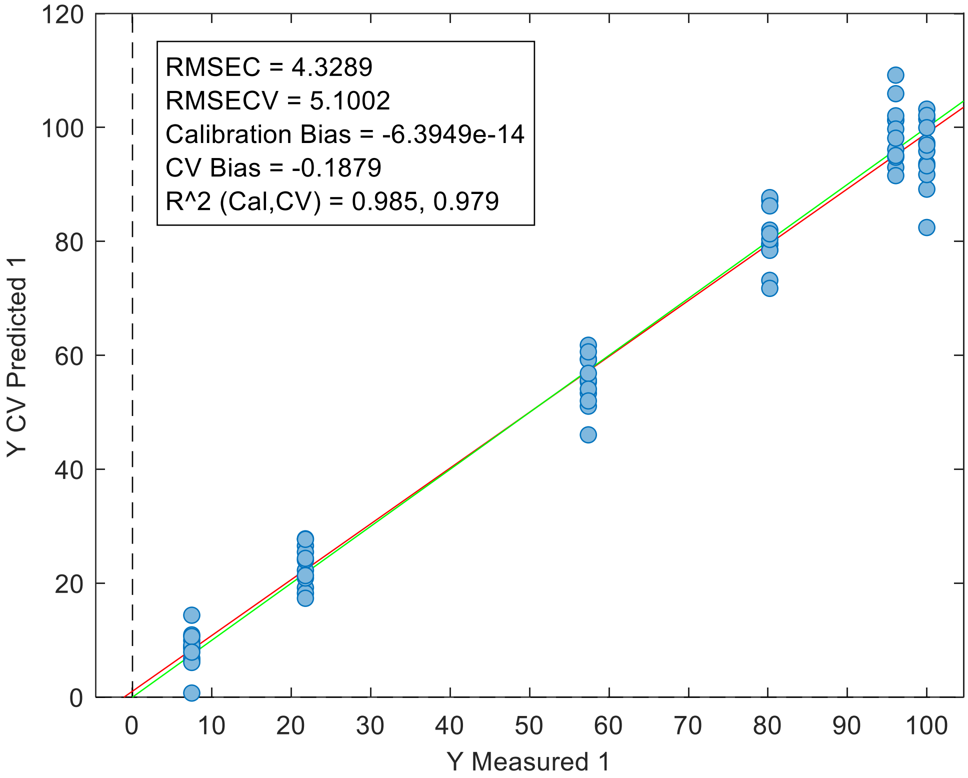

Figure S2. The results of the viability determination for A375 cells exposed to the various doses of oxaliplatin, obtained using MLR based on PARAFAC scores as inputs. The cross-validation results of MLR@PARAFAC are plotted vs. MTT results. The green line represents an ideal fit, whereas the red one – obtained in the experiment. MLR@PARAFAC – MLR based on PARAFAC scores of components 1-5.

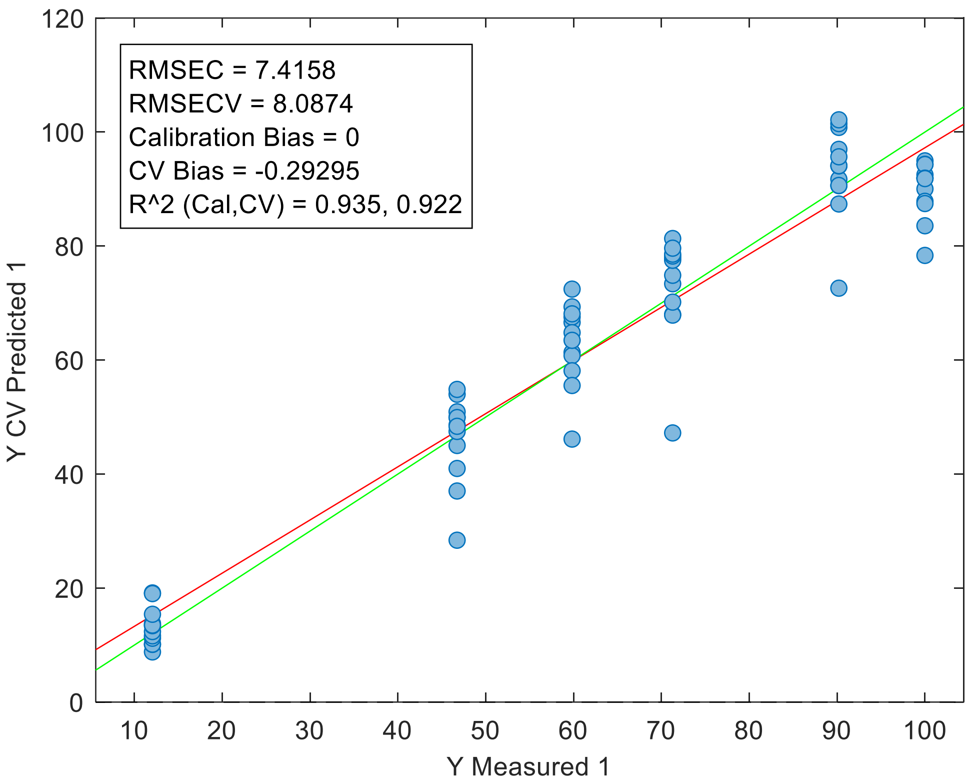

Figure S3. The results of the viability determination for HaCaT cells exposed to the various doses of oxaliplatin, obtained using MLR based on PARAFAC scores as inputs. The cross-validation results of MLR@PARAFAC are plotted vs. MTT results. The green line represents an ideal fit, whereas the red one – obtained in the experiment. MLR@PARAFAC – MLR based on PARAFAC scores of components 1-5.
